# Adherence to the transfer recommendations of the German Trauma Society in severely injured children: a retrospective study from the TraumaRegister DGU

**DOI:** 10.1038/s41598-023-39335-8

**Published:** 2023-07-27

**Authors:** Felix Marius Bläsius, Markus Laubach, Rolf Lefering, Frank Hildebrand, Hagen Andruszkow

**Affiliations:** 1 Department of Orthopaedics, Trauma and Reconstructive Surgery, University Hospital RWTH Aachen, Pauwelsstraße 30, 52074 Aachen, Germany; 2 Centre for Regenerative Medicine, Institute of Health and Biomedical Innovation, Queensland University of Technology, Brisbane, QLD 4059 Australia; 3 Institute for Research in Operative Medicine (IFOM), Faculty of Health, Witten/Herdecke University, Ostmerheimer Straße 200, Haus 38, 51109 Cologne, Germany

**Keywords:** Paediatric research, Outcomes research

## Abstract

Particularly for pediatric trauma patients, it is of utmost importance that the right patient be treated in the right place at the right time. While unnecessary interhospital transfers must be avoided, the decision against transfer should not lead to higher complication rates in trauma centers without added pediatric qualifications. We therefore identified independent predictive factors for an early transfer of severely injured patients and compared these factors with the current transfer recommendations of the German Trauma Society. Additionally, the quality of the self-assessment based on the mortality of children who were not transferred was evaluated. A national dataset from the TraumaRegister DGU^®^ was used to retrospectively identify factors for an early interhospital transfer (< 48 h) to a superordinate trauma center. Severely injured pediatric patients (age < 16 years) admitted between 2010 and 2019 were included in this analysis. Adjusted odds ratios (OR) with 95% confidence intervals (CI) for early transfer were calculated from a multivariable model. Prognostic factors for hospital mortality in non-transferred patients were also analyzed. In total, 6069 severely injured children were included. Of these, 65.2% were admitted to a Level I trauma center, whereas 27.7% and 7.1% were admitted to Level II and III centers, respectively. After the initial evaluation in the emergency department, 25.5% and 50.1% of children primarily admitted to a Level II or III trauma center, respectively, were transferred early. Statistically significant predictors of an early transfer were: Serious traumatic brain injury (OR 1.76, 95% CI 1.28–2.43), Injury severity score (ISS) ≥ 16 points (ISS 16–24: OR 2.06, 95% CI 1.59–2.66; ISS 25–33: OR 3.0, 95% CI 2.08–4.31; ISS 34–75: OR 5.42, 95% CI 3.0–9.81, reference category: ISS 9–15), age < 10 years (age 0–1: OR 1.91, 95% CI 1.34–2.71; age 2–5: 2.04, 95% CI 1.50–2.78; age 6–9: 1.62, 95% CI 1.23–2.14; reference category: age 10–15). The most important independent factor for mortality in non-transferred patients was age < 10 years (age 0–1: 5.35, 95% CI 3.25–8.81; age 2–5: 2.46, 95% CI 1.50–4.04; age 6–9: OR 1.7, 95% CI 1.05–2.75; reference category: age 10–15). Knowing the independent predictors for an early transfer, such as a young patient's age, a high injury severity, serious traumatic brain injury (TBI), may improve the choice of the appropriate trauma center. This may guide the rapid decision for an early interhospital transfer. There is still a lack of outcome data on children with early interhospital transfers in Germany, who are the most vulnerable group.

## Introduction

With a focus on the quality of care, requirements for human and structural resources for Level I trauma centers treating severely injured children have been formulated in Germany. Therefore, the S3-guidelines for the treatment of polytrauma and the severely injured^1^, as well as the Whitebook Medical Care of the Severely Injured (3rd Revision) of the German Trauma Society^2^, emphasize the relevance of early recognition of pediatric patients who require special treatment in a Level I trauma center. In this context, specific factors requiring advanced management, such as severe injuries (e.g., traumatic brain injury [TBI], pelvic trauma) or an impaired general status (e.g., ICU treatment > 48 h), have been defined as indications for a transfer from Level II and III trauma centers to centers with the highest treatment capability (Level I center with added pediatric qualifications). Although the American College of Surgeons Committee on Trauma (ACS-COT) has defined clear goals for over-triage (25–35%) and under-triage (< 5%)^3^ for severely injured patients, several studies have reported deficiencies regarding the accuracy of pre-hospital and early emergency room triage criteria in severely injured children^4,5^. These shortcomings could lead to delayed or missed interhospital transfers to Level I trauma centers and may increase the risk of adverse outcomes. In contrast, unnecessary transport may also endanger the patient.

For this reason, early, and reliable identification of pediatric trauma patients at risk is desirable to promptly initiate early interhospital transfer or even improve the preclinical triage of pediatric trauma patients who could benefit from a primary admission or a transfer to a high-level center if the mission tactics allow (e.g., the timely reachability of a Level I center). This raised the first question of which criteria increased the likelihood of an early transfer from Level II or III centers to a Level I trauma center in the last decade in Germany and if these criteria correspond to the recommendations mentioned above. As a second research question, we evaluated whether the mortality of non-transferred children was comparable to that of patients treated in Level I centers.

Therefore, the primary objective of this study was to identify independent predictive factors for an early transfer of severely injured patients and, using a multivariable regression approach (Model A), compare the identified predictive factors with the current transfer recommendations of the German Trauma Society for pediatric trauma patients. A secondary study objective was to evaluate the quality of the self-assessment based on the mortality of children who were not transferred using a second multivariable regression model (Model B).

## Methods

The TraumaRegister DGU^®^ (TR-DGU) of the German Trauma Society was founded in 1993 to create a multi-center database for the pseudonymized, standardized documentation of severely injured patients for quality assurance and research^6^. Participating hospitals are primarily located in Germany (90%), but a rising number of hospitals from other countries have begun to contribute data as well, including Austria, Belgium, Finland, Luxembourg, Slovenia, Switzerland, the Netherlands, and the United Arab Emirates. Approximately 28,000 cases from almost 700 hospitals are currently entered into the database annually. Participation in the TR-DGU is voluntary. For hospitals associated with TraumaNetzwerk DGU^®^, however, the entry of at least a basic data set is obligatory for reasons of quality assurance. Documentation included detailed information on demographics, injury patterns, comorbidities, pre- and in-hospital management, course of care while in the intensive care unit, relevant laboratory findings (including data on transfusions), and the clinical outcomes of each individual. The inclusion criteria for the TR-DGU are a) admission to a hospital via the emergency room with subsequent intensive care or entrance to a hospital with vital signs and b) death prior to admission to the ICU.

The infrastructure for documentation and data management is provided by the AUC—Academy for Trauma Surgery (AUC—Akademie der Unfallchirurgie GmbH), a company affiliated with the German Trauma Society. Scientific leadership is provided by the Committee on Emergency Medicine, Intensive Care, and Trauma Management (Sektion NIS) of the German Trauma Society. Participating hospitals submit pseudonymized data into a central database via a web-based application. Patients were included in the registry on the basis of informed consent, which was obtained from the patients and/or their legal guardian(s). The database inclusion, methods, analyses, and interpretation were carried out in accordance with the EU General Data Protection Regulation, the Federal Data Protection Act Germany, and the Declaration of Helsinki. Scientific data analysis is approved according to a peer review procedure established in the publication guidelines of the TR-DGU.

This study is consistent with the publication guidelines of the TR-DGU and is registered and approved as TR-DGU project ID 2020-018 by the TR-DGU Review Board (https://www.traumaregister-dgu.de/) and the Committee on Emergency Medicine, Intensive Care, and Trauma Management of the German Trauma Society.

### Trauma care in Germany

Three levels of trauma centers are available in Germany. Level I trauma centers are considered the supra-regional maximum care provider. Germany is divided into 52 regional trauma networks (TraumaNetzwerk DGU^®^). The detailed specifications for each level of care are published in the Whitebook Medical Care of the Severely Injured and in former studies^2,7^.

#### Transfer criteria

The independent transfer criteria evaluated in this study were compared to the transfer recommendations from the Whitebook Medical Care of the Severely Injured (3rd Revision) of the German Trauma Society^2^. Severely injured children should be admitted to a Level I center with added pediatric expertise if such a center is available within 30 min. Otherwise, children should be resuscitated in the nearest trauma center and secondarily transferred to a Level I center with added pediatric expertise according to the following criteria:

- GCS ≤ 12 points.
- AIS_Thorax_ ≥ 3 points and/or lung contusion.
- AIS_Abdomen_ ≥ 3 points with organ injury.
- Pelvic fracture or fractures of ≥ 2 long bones of the lower extremity.
- Intensive care stay ≥ 24 h.
- ISS ≥ 16 points.

### Inclusion criteria

The present retrospective observational study included the following patients from the TR-DGU:

- Date of admission from January 2010 to December 2019 (10 years).
- Treatment in a German trauma center.
- Trauma team activation/resuscitation team.
- Maximum Abbreviated Injury Scale (MAIS) ≥ 3 points.
- Age < 16 years.
- Primary admissions from the scene of injury.

### Definitions

Patients younger than 16 years old were defined as children. The Abbreviated Injury Scale (AIS, Version 2005/Update 2008, Association for the Advancement of Automotive Medicine [AAAM], Barrington, IL) was used as a global system for injury coding and severity classification. The severity of injuries was recorded according to the AIS as 1 (minor), 2 (moderate), 3 (severe, not life-threatening), 4 (serious, life-threatening), 5 (critical, survival uncertain), and 6 (maximum, currently untreatable). The MAIS is the highest AIS code in a polytraumatized patient. It was calculated for each patient, and overall injury severity was calculated by ISS as described by Baker et al.^8^. Patients were categorized into four ISS groups (9–15, 16–24, 25–33, 34–75) according to the AAAM^9^. A serious traumatic brain injury was defined as AIS_Head_ ≥ 3 points. Early transfers were defined as transfers within 48 h after admission. Weekend admissions were defined as admissions from Friday to Sunday. Nighttime admissions were from 6 pm to 6 am. Multiple organ failure (MOF) was diagnosed according to the Sequential Organ Failure Assessment (SOFA), in which 3 or 4 points for an organ were considered organ failure^10^. Sepsis was based on the sepsis-2 definition because it could be applied to patients before and after the last revision (sepsis-3 definition)^11^.

### Statistics

Categorical variables are presented as percentages only if the underlying total is obvious. Continuous values are presented as mean with standard deviation (SD) or median with quartiles, if applicable. Differences in categorical and continuous variables were evaluated with the Chi-square test and Mann–Whitney U test, respectively. The significance level was set at 0.05 (two-sided). The statistical trend level was set at 0.10 (two-sided). Numbers are given in descending order of trauma centers (Level I, Level II, Level III, *p*-value). All statistical analyses were performed using the Statistical Package for the Social Sciences (SPSS 25.0; IBM Inc., Armonk, NY, USA).

#### Multivariable logistic regression models

We applied two multivariable logistic regression models to identify predictors for the dependent endpoints: "early transfer" (Model A) and "hospital mortality" (Model B). The results were reported as adjusted odds ratios (AOR) with a 95% CI.

Model A was calculated to identify predictor variables for an early transfer of severely injured children to a superordinate trauma center and to enable the comparison with the current transfer recommendation of the German Trauma Society. The model included the following variables:

- ISS (9–15/16–24/25–33/34–75).
- Admission on weekends.
- Admission during the night.
- Blood transfusion.
- Pre-hospital CPR.
- Pre-hospital endotracheal intubation.
- AIS ≥ 3 for relevant body regions (Thorax, Abdomen, Head, Pelvic/Extremities).
- Age groups (0–1/2–5/6–9/10–15).
- Level III treatment.

This model included only patients admitted to Level II and III hospitals.

Model B was calculated to evaluate the quality of the self-assessment based on the mortality of children who were not transferred. The model included the following variables to adjust for possible confounders:

- RISC II score (as a combination of 13 known predictors).
- Hospital level of care (I–III).
- Mode of transportation (ground/helicopter).
- Age groups of children (0–1/2–5/6–9/10–15).

This study excluded early transfer-out patients since their outcome was considered unclear. Since the treatment level and the transport mode are structural factors that interact, we included both in our analysis. Thus, we could exclude the bias that severely injured children are predominately transported by helicopter emergency medical service (HEMS) to Level I centers, which could have falsified our results. The oldest age group (10–15 years) and Level I trauma centers were chosen as the reference categories.

#### Assessment of mortality risk

The Revised Injury Severity Classification version 2 (RISC II) score was developed and validated for risk of death prediction based on TR-DGU data^12^ and used for adjustments in multivariate analyses before^7,13^. The score includes 13 variables, such as injury pattern and severity, physiology on admission, the American Society of Anesthesiologists (ASA) physical status classification system, motor function, pupil status, and the injury mechanism. RISC II scores were used to adjust the observed mortality rates by calculating the ratio of observed vs. expected mortality rate standardized mortality ratio (SMR). The SMR values were given with 95% confidence intervals (CI) based on the respective CI of the observed mortality. Student's t-test was used to evaluate SMR differences.

### Ethics approval and consent to participate

Patients were included in the registry on the basis of informed consent, which was obtained from the patients and/or their legal guardian(s). The database inclusion, all methods, analyses, and interpretation were carried out in accordance with the EU General Data Protection Regulation, the Federal Data Protection Act Germany, and the Declaration of Helsinki. This study is consistent with the publication guidelines of the TR-DGU and is registered and approved as TR-DGU project ID 2020-018 by the TR-DGU Review Board (https://www.traumaregister-dgu.de/) and the Committee on Emergency Medicine, Intensive Care, and Trauma Management of the German Trauma Society.

## Results

**Figure 1:**
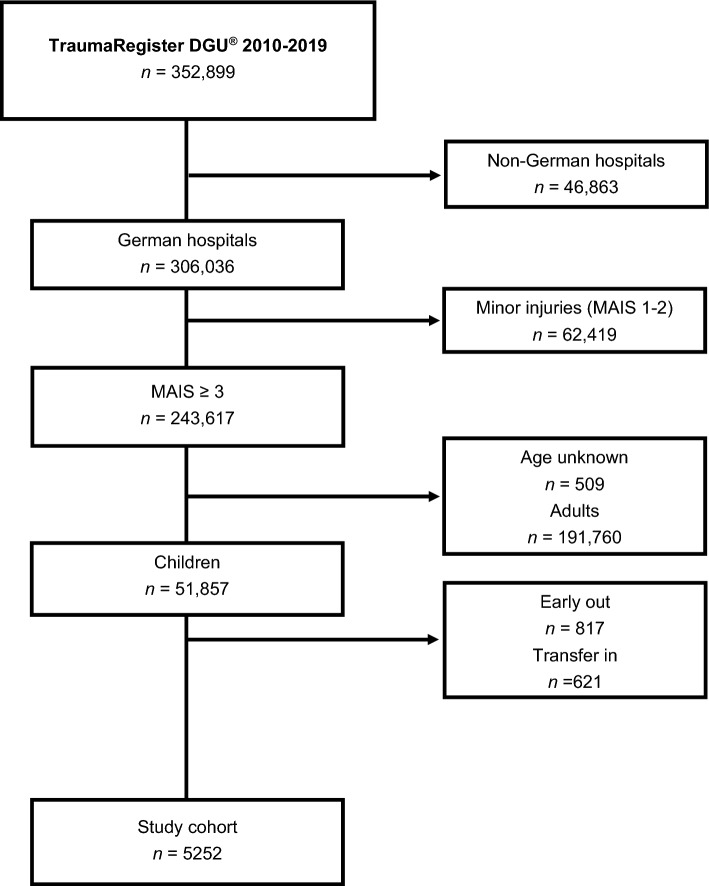
Study flow chart illustrating the selection of patients.

**Figure 2:**
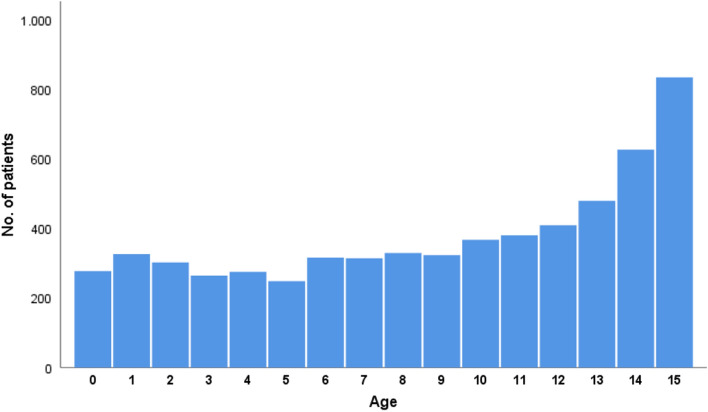
Histogram showing the age distribution of the patients.

A total of 6069 pediatric trauma patients (Level I: 3954 [65.2%], Level II: 1684 [27.7%], and Level III: 431 [7.1%]) were included. The mean age was 8.9 ± 5.1 years. Figure 1 shows the inclusion and exclusion process, and Fig. 2 shows the age distribution.

### Patient characteristics

Children treated in Level I trauma centers were younger than children treated in Level II and Level III centers. Sex ratios were comparable in each group. More frequent use of medical imaging was observed in the Level I group compared to centers of lower treatment levels (each p < 0.001). ISS was highest in Level I trauma centers compared to Level II and III centers (median [quartiles]; 17 [11–25], 16 [10–21], 14 [10–18], respectively, p < 0.001). The proportion of patients with serious TBI (AIS_Head_ ≥ 3; 56.3%, 41.9%, 37.6%; p < 0.001) and unconsciousness (GCS < 9; 28.1%, 16.4%, 9.2%, p < 0.001) was most frequently recorded in Level I centers. Table 1 summarizes the baseline characteristics.

**Table 1:** Patient and hospital characteristics by trauma center level.

	Level I	Level II	Level III	Total	*p*-value
Primary admissions (n)	3 954	1 684	431	6 069	
Age (years)	8.7 (4.8)	9.2 (4.9)	9.3 (5.1)	8.9 (5.1)	< 0.001
Male (%)	2456 (62.2%)	1045 (62.1%)	282 (65.4%)	3783 (62.4%)	0.401
Penetrating trauma (%)	101 (2.7%)	55 (3.4%)	9 (2.3%)	165 (2.9%)	0.241
Injury mechanisms (%)					< 0.001
Car	502 (12.9%)	193 (11.7%)	47 (11.4%)	742 (12.5%)	
Motorcyclist	175 (4.5%)	90 (5.5%)	32 (7.8%)	297 (5.0%)	
Bicycle	548 (14.1%)	279 (17.0%)	75 (18.2%)	902 (15.2%)	
Pedestrian	864 (22.2%)	316 (19.2%)	74 (18.0%)	1254 (21.1%)	
Hight fall > 3 m	736 (18.9%)	251 (15.3%)	40 (9.7%)	1027 (17.3%)	
Low fall < 3 m	493 (12.7%)	257 (15.6%)	78 (18.9%)	828 (13.9%)	
Others	575 (14.8%)	258 (15.7%)	66 (16.0%)	899 (15.1%)	
Times (min mean/median, SD)					
On scene	28/25 (17)	22/20 (14)	18/18 (10)	27/25 (17)	< 0.001
From accident to hospital	63/60 (28)	55/50 (30)	52/45 (39)	60/55 (30)	< 0.001
Emergency room	67/50 (61)	77/59 (63)	90/69 (63)	72/52 (62)	< 0.001
GCS < 9 pts. (%)	1011 (28.1%)	250 (16.4%)	35 (9.2%)	1296 (23.5%)	< 0.001
HEMS transport (%)	1465 (38.7%)	140 (8.6%)	11 (2.7%)	1616 (27.8%)	< 0.001
Admission during night time (%)	1548 (39.2%)	671 (39.8%)	152 (35.3%)	2371 (39.1%)	0.217
Weekend admissions (%)	1776 (44.9%)	742 (44.1%)	183 (42.5%)	2701 (44.5%)	0.567
ER diagnostics					
FAST sonography (%)	3480 (88.7%)	1429 (85.9%)	361 (84.1%)	5270 (87.6%)	0.001
X-ray (%)	1394 (35.5%)	445 (26.8%)	117 (27.3%)	1956 (32.5%)	< 0.001
CT scan (%)	3272 (82.8%)	1211 (71.9%)	275 (63.8%)	4758 (78.4%)	< 0.001
LOS (days) median (IQR)	9 (5–16)	6 (1–11)	1 (1–7)	8 (3–14)	< 0.001
ICU stay (%)	3411 (86.3%)	1171 (69.5%)	217 (50.3%)	4799 (79.1%)	< 0.001
ICU LOS (days) median (IQR)	2 (1–6)	1 (0–3)	0 (0–1)	2 (1–4)	< 0.001
Early transfer out (%)	172 (4.4%)	429 (25.5%)	216 (50.1%)	817 (13.5%)	< 0.001
Discharged home (%)	2580 (65.3%)	983 (58.4%)	178 (41.3%)	3741 (61.6%)	< 0.001
Died in hospital (%)	333 (8.4%)	94 (5.6%)	7 (1.6%)	434 (7.2%)	< 0.001
ISS (points)	20.2/17 (12.0)	17.7/16 (10.0)	16.3/14 (9.3)	19.2/16 (11.4)	< 0.001
ISS ≥ 16 (%)	2441 (61.7%)	894 (53.1%)	209 (48.5%)	3544 (58.4%)	< 0.001
Injured body regions (AIS ≥ 3					
Head (%)	2227 (56.3%)	705 (41.9%)	162 (37.6%)	3094 (51.0%)	< 0.001
Thorax (%)	1086 (27.5%)	485 (27.2%)	103 (23.9%)	1647 (27.1%)	0.286
Abdomen (%)	482 (12.2%)	285 (16.9%)	68 (15.8%)	835 (13.8%)	< 0.001
Pelvic/extremities (%)	1175 (29.7%)	494 (29.3%)	142 (32.9%)	1811 (29.8%)	0.329

Continuous variables are presented as mean/median with standard deviation (SD).

### Early transfers

**Figure 3:**
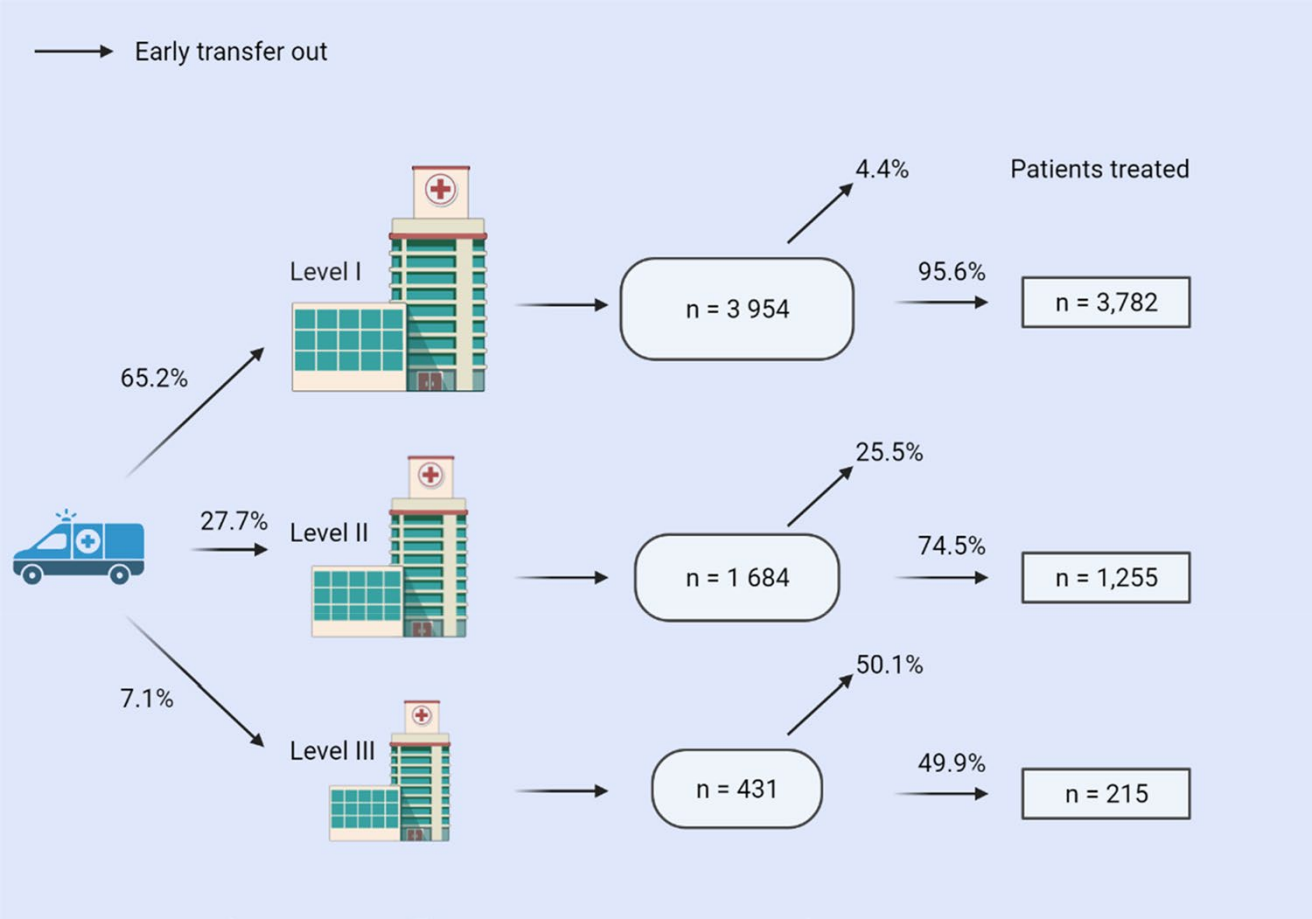
Illustration of pre-hospital triage, early transfers, and non-transferred patients (Created with BioRender.com).

In the case of an interhospital transfer, patients initially admitted to a Level II or III center were predominately (> 90% of all cases) transferred to a Level I trauma center. Thus, 50.1% and 25.5% of children treated in a Level III or II center, respectively, were transferred within 48 h after the initial emergency assessment (Fig. 3).

### Predictors of an early transfer

Because of the high rates of early interhospital transfers from Level II and III centers, we performed a multivariable regression analysis to identify independent predictors for transfers only in children admitted to these centers. Table 2 summarizes the results from Model A, with ORs for an early transfer. The model showed a nearly fourfold increased probability of an early transfer (OR 3.80) if the patient was primarily admitted to a Level III trauma center compared to admission to a Level II center. Moreover, younger children < 10 years of age also showed an increased probability for an early transfer (ORs > 1.6, *p* < 0.05 for each age group, Table 2) compared to the reference category (age 10–15 years). Interestingly, serious TBI (AIS_Head_ ≥ 3) increased the probability of early transfer (OR 1.76). In contrast, a severe trauma of the thorax (OR 0.54), abdomen (OR 0.64), or extremities (OR 0.43) did not, after adjustment for overall severity (ISS). A preclinical endotracheal intubation (OR 0.47) and a pre-hospital traumatic cardiac arrest (OR 0.45) also did not favor an early transfer (Table 2).

**Table 2:** Model A.

	Adjusted odds ratio (AOR)	95% CI for OR	*p*-value
Level III	3.80	2.95–4.90	< 0.001
Age (years; reference: 10–15)			< 0.001
0–1	1.91	1.34–2.71	0.001
2–5	2.04	1.50–2.78	< 0.001
6–9	1.62	1.23–2.14	0.001
AIS_Head_ ≥ 3	1.76	1.28–2.43	0.001
AIS_Thorax_ ≥ 3	0.54	0.39–0.74	< 0.001
AIS_Abdomen_ ≥ 3	0.64	0.44–0.94	0.022
AIS_Pelvic/Extremitites_ ≥ 3	0.43	0.31–0.60	< 0.001
Preclinical endotracheal intubation	0.47	0.33–0.68	< 0.001
Pre-hospital cardiac arrest, CPR	0.45	0.24–0.84	0.012
Blood transfusion	1.11	0.63–1.94	0.723
Admission during the night	1.02	0.81–1.27	0.891
Admission on weekend (Fr.-Su.)	1.03	0.83–1.28	0.782
ISS (points; reference 9–15)			< 0.001
16–24	2.06	1.59–2.66	< 0.001
25–33	3.00	2.08–4.31	< 0.001
34–75	5.42	3.0–9.81	< 0.001

Multivariable binary regression analysis with “early transfer” as dependent variable (*n* = 2115). Only primary admitted patients from Level II and III hospitals have been included. The analysis includes both, transferred and non-transferred patients.

Nagelkerke’s R^2^ = 0.236.

### Mortality of non-transferred children

Mortality analysis was limited to cases not transferred to another center, as no sufficient data about the outcome was available. The observed hospital mortality tended to be higher than expected mortality in Level II and Level III centers compared to the expected mortality (Table 3). However, there was no significant difference in SMRs between Level I (1.00, 95% CI [0.90–1.10]), Level II (1.10, 95% CI [0.89–1.32]), and Level III (1.05, 95% CI [0.29–1.82]) trauma centers (*p* = 0.36, Table 3). The hospital level of care was also evaluated with a multivariable logistic regression analysis (Model B). Our analysis showed increased mortality with decreasing patient age (Table 4). The youngest patient group (0–1 years) had the highest risk of death compared to the oldest age group (OR 5.35). The hospital level of care (II and III) showed a discrete but not significant increased risk of death.

**Table 3:** Observed and expected mortality rates using the RISC II as well as the calculated standardized mortality rates (non-transferred patients).

	Level I	Level II	Level III	*p*-value
Primary admitted, with outcome (n)	3 782	1 255	215	
Expected mortality based on RISC II (%)	8.8%	6.8%	3.1%	< 0.001
Observed mortality (% with 95% CI)	8.8 (7.9–9.7)	7.5 (6.0–8.9)	3.3 (0.9–5.6)	< 0.001
SMR (95% CI)	1.00 (0.90–1.10)	1.10 (0.89–1.32)	1.05 (0.29–1.82)	0.36

**Table 4:** Model B.

	Adjusted odds ratio (OR)	95% CI for OR	*p*-value
RISC II score	0.33	0.30–0.36	< 0.001
Age (years; reference 10–15)			< 0.001
0–1	5.35	3.25–8.81	< 0.001
2–5	2.46	1.50–4.04	< 0.001
6–9	1.70	1.05–2.75	0.032
HEMS transport	0.83	0.57–1.20	0.313
Level of care (reference: Level I)			0.282
Level II	1.45	0.92–2.30	0.113
Level III	1.22	0.29–5.03	0.789

Multivariable binary logistic regression analysis with "mortality" as the dependent variable. Primary admitted patients who were not transferred from all three levels were included. Only patients transferred out early were excluded since their outcome was considered uncertain (*n* = 5252).

Nagelkerke’s R^2^ = 0.739.

## Discussion

In this retrospective study, we demonstrated high early transfer (< 48 h) rates of 25–50% in children initially treated in Level II and III trauma centers. If the trauma centers decided that pediatric patients did not need to be transferred, the non-transferred patients showed a minor trend toward higher standardized mortality rates in these centers. However, this effect was insignificant, even if adjusted for further predictors beyond those included in the RISC II.

Identifying severely injured patients with a high probability of an early interhospital transfer or who should be initially admitted to a Level I center remains a major challenge. Despite all research efforts, existing scores for clinical decision-making aiming to admit severely injured patients to the most appropriate center or to identify patients who would benefit from an early transfer show high variability and insufficient sensitivity and specificity^14^. This might result in a non-achievement of the under-triage (< 5%) and over-triage (25–35%) goals of the ACS-COT^3^. Accordingly, our data showed a high proportion of early interhospital transfers after admission and initial emergency assessment, with the associated risk of delaying potentially life-saving interventions (e.g., TBI, vascular injuries). Several factors may explain the high early transferal rates of severely injured children:

1. Pre-hospital triage of children and adolescents is more difficult than for adults, as the risk of misjudgment of both injury severity and cardiopulmonary stability is high due to the special features of pediatric patients. In particular, the distinct compensation capabilities of pediatric patients often lead to the phenomenon of a hemorrhagic shock remaining occult for a long time and sudden cardiopulmonary instability occurring.
2. Communication is limited; therefore, early assessment may be difficult, particularly in the youngest patients.
3. Tactical considerations prioritizing the resuscitation of unstable trauma patients in the nearest center and accepting a secondary transfer to a superordinate trauma center may play a role. This is mainly supported by our data showing that an overall critical condition of the patient (e.g., pre-hospital intubation and traumatic cardiac arrest) reduces the chance of an early transfer (Table 2). This approach is consistent with the internationally implemented Advanced Trauma Life Support (ATLS) recommendations and national guidelines in Germany^1^.

Moreover, our multivariable analysis (Model A) demonstrated several factors influencing the decision to transfer severely injured children to a superordinate trauma center. Low patient age, high ISS scores, and serious TBI independently predicted early transfer (Table 2). The factors increasing the probability of early transfer identified in our study coincide with the transfer criteria listed in the Whitebook of the German Trauma Society. According to the Whitebook^2^, transfer to a Level I center with added pediatric qualifications is recommended in the presence of an ISS ≥ 16 and serious TBI. The importance of these variables has already been described in international studies, since almost all international guidelines and recommendations consider these criteria for transfer^15^. Also, in the case of serious injuries to specific other body regions (abdomen, thorax, pelvis), a secondary transfer should be considered, according to the Whitebook. Interestingly, our study did not find these injuries to be independent predictive factors. This could be due to several factors.

On the one hand, according to current standards of care, resuscitation is first performed, and the definitive care of complex orthopedic injuries is postponed to a later date. Furthermore, the observation that these injuries do not predict an early transfer may be attributed to sufficient surgical expertise for the early resuscitation phase across the three trauma center levels. In Germany, the certification criteria require that surgical expertise (visceral and orthopedic trauma) and an ATLS or European Trauma Course (ETC) trained surgeon be available 24/7, even at the lowest certification level (Level III). This ensured adequate emergency care for children with serious abdominal, thoracic, or pelvic trauma. This process is also supported by our results. We were able to show that about 50% of the children who were treated in a level III center were subsequently transferred. Transportation to a Level I trauma center with added pediatric expertise is, therefore, likely to maintain the function of the extremity, to guarantee the best outcome and to ensure a safe transportation after initial resuscitation. However, our study did not investigate later transfers (> 48 h after admission). Accordingly, only the early transports can be evaluated in this work. Furthermore, the timing of admission (weekends and nights) was not an independent variable predisposing to an early transfer; it could be attributed to the 24/7 availability of the disciplines mentioned above.

The treating trauma center decided whether or not to transfer a child. We analyzed the reliability of this decision by targeting the mortality of non-transferred patients. Fortunately, the early admission triage of the Level II and Level III trauma centers was valid and did not result in statistically significant higher SMRs in the completion of treatment without secondary transfer. However, we could show that the children who were not transferred tended toward higher mortality rates in Level II and III trauma centers (AORs 1.45 and 1.22, respectively) but missed the level of significance. Nevertheless, we could show significant differences in a previous study for 2013–2017, supporting a potential outcome benefit by Level I treatment^7^. In the present study, the multivariable analysis performed using the RISC II method confirmed the results of the SMR comparison. The evidence for lower mortality from treatment in Level I centers remains intermediate to weak, depending on the applied statistical method and the confounder considered.

In summary, the transfer criteria, according to the Whitebook, are well implemented. Unfortunately, outcome data regarding secondary transferred patients are lacking. Merging individual data from the centers and assigning them to a patient continues to be impossible. Thus, we could not further characterize this vulnerable early transferred patient group.

Overall, there are conflicting positions in the international literature on whether specialized trauma centers, the trauma center level, or the caseload of a trauma center independently influence the outcomes of severely injured children. Existing studies are hardly comparable due to the different national trauma center certification systems; therefore, little evidence is available. In 2015, Sathya et al. demonstrated in a large U.S. study that high-volume and ACS-certified pediatric trauma centers showed lower mortality risks than low-volume and adult/mixed trauma centers^16^. The authors adjusted their multivariate analyses for confounders (patient and injury characteristics).

In contrast, in 2001, Sherman et al. also demonstrated a lower mortality risk in children treated in a Level I or Level I trauma center with added qualifications in pediatrics compared to pediatric trauma centers or Level II trauma centers in a US cohort of over 16,000 children^17^. In contrast to Sathya et al., Sherman et al. performed an adjustment according to the TRISS method. No studies from Germany or nations with comparable trauma center certification systems are available. A comparison with studies from the US did not seem plausible due to the lack of comparability of the trauma center certification systems and shortcomings using the TRISS method in analyses in the German trauma population^12^.

Nonetheless, reasons for the high rates of secondary transfers should be further explored (e.g., access to high-level centers or potential inaccuracies in preclinical triage) and refined. In addition, it is imperative that we observe and study the group of secondarily transferred patients in more detail in the future to identify potential outcome differences and to develop preclinical triage and transfer criteria.

## Strengths and limitations

Regarding the limitations, our multivariable regression models could only be based on those confounders documented in the registry. Furthermore, it is impossible to link the treatment phases of a patient transferred from different hospitals with sufficient precision. Thus, it was not possible to directly compare the outcome of transferred patients to those who received definite care in the first hospital. Another limitation is the discrepancy between our inclusion criteria (age < 16 years) and the patient group defined by the Whitebook (age < 12 years). However, we do not believe that this circumstance negatively influenced the conclusions of our study. Besides the focus on severely injured patients (MAIS ≥ 3), another strength of our study is the sample size of the analyzed cohort and the data quality of the registry. However, the study was limited by its retrospective nature, as we had to rely on the accuracy of the recorded data. Finally, this is the first study to investigate predictors for early interhospital transport and independent predictors for mortality in non-transferred severely injured pediatric patients in Germany.

## Conclusion

Overall, national, and international transfer criteria were implemented. Furthermore, there is still a lack of outcome data on children with early interhospital transfers in Germany, who are the most vulnerable group. Characterizing this patient group may be critical for reliably reducing and identifying severely injured trauma patients who need to be transferred to another trauma center.

## Data Availability

The sensitive data presented in this study are available from a third party, the AUC—Academy for Trauma Surgery (AUC—Akademie der Unfallchirurgie GmbH), but restrictions apply to the availability of these data, which were used under license for the current study, and so are not publicly available. Data are however available from the first author F.M.B. upon reasonable request and with permission of AUC (AUC—Akademie der Unfallchirurgie GmbH, Emil-Riedel-Straße 5, 80538 München, Deutschland, Email: support-tr@auc-online.de).

## Abbreviations

AIS: Abbreviated Injury Scale
AUC: Academy for Trauma Surgery
DGU: German Trauma Society
GEMS: Ground Emergency Medical Services
HEMS: Helicopter Emergency Medical Services
ICU: Intensive care unit
ICU LOS: Intensive care unit length of stay
IQR: Interquartile range
ISS: Injury Severity Score
LOS: Length of stay
MAIS: Maximum AIS
NTDB: National Trauma Data Bank
OR: Odds ratio
PICU: Pediatric intensive care unit
RISC II: Revised Injury Severity Classification Score II
SD: Standard deviation
TR-DGU: TraumaRegister DGU^®^
