# Author Correction: Adherence to the transfer recommendations of the German Trauma Society in severely injured children: a retrospective study from the TraumaRegister DGU

**DOI:** 10.1038/s41598-023-43529-5

**Published:** 2024-01-26

**Authors:** Felix Marius Bläsius, Markus Laubach, Rolf Lefering, Frank Hildebrand, Hagen Andruszkow

**Affiliations:** 1 Department of Orthopaedics, Trauma and Reconstructive Surgery, University Hospital RWTH Aachen, Pauwelsstraße 30, 52074 Aachen, Germany; 2 Centre for Regenerative Medicine, Institute of Health and Biomedical Innovation, Queensland University of Technology, Brisbane, QLD 4059 Australia; 3 Institute for Research in Operative Medicine (IFOM), Faculty of Health, Witten/Herdecke University, Ostmerheimer Straße 200, Haus 38, 51109 Cologne, Germany

Correction to: *Scientific Reports* https://doi.org/10.1038/s41598-023-39335-8, published online 27 July 2023

The original version of this Article contained errors in the Reference list.

Reference 2 was erroneously cited in the body of the text. In addition, Reference 2 was incorrectly given as:

Schoeneberg, C. *et al.* TraumaNetwork, Trauma Registry of the DGU(R), Whitebook, S3 Guideline on Treatment of Polytrauma/Severe Injuries—An Approach for Validation by a Retrospective Analysis of 2304 Patients (2002–2011) of a Level 1 Trauma Centre. *Zentralbl Chir* 142, 199–208. 10.1055/s-0033-1360225 (2017).

The correct reference is listed below.

German Trauma Society. Whitebook Medical Care of the Severely Injured 2nd revised and updated edition. Available online: https://www.dgu-online.de/fileadmin/dgu-online/Dokumente/6._Versorgung_und_Wissenschaft/Qualit%C3%A4t_und_Sicherheit/2019_DGU-Weissbuch_Schwerverletztenversorgung_3._Auflage_FINAL.PDF (Accessed 17 Jan 2021).

As a result, the below References were omitted and are now listed as References 8, 10, 11, 12, 14 and 17.

Baker, S. P., O'Neill, B., Haddon Jr, W. & Long, W. B. The injury severity score: A method for describing patients with multiple injuries and evaluating emergency care. *J. Trauma* **1974**, *14*, 187–196.

Vincent, J.L.; Moreno, R.; Takala, J.; Willatts, S.; De Mendonca, A.; Bruining, H.; Reinhart, C.K.; Suter, P.M.; Thijs, L.G. The SOFA (Sepsis-related Organ Failure Assessment) score to describe organ dysfunction/failure. On behalf of the Working Group on Sepsis-Related Problems of the European Society of Intensive Care Medicine. *Intensive Care Med* **1996**, *22*, 707-710, doi:10.1007/BF01709751.

Bone, R.C.; Balk, R.A.; Cerra, F.B.; Dellinger, R.P.; Fein, A.M.; Knaus, W.A.; Schein, R.M.; Sibbald, W.J. Definitions for sepsis and organ failure and guidelines for the use of innovative therapies in sepsis. The ACCP/SCCM Consensus Conference Committee. American College of Chest Physicians/Society of Critical Care Medicine. *Chest* **1992**, *101*, 1644-1655, doi:10.1378/chest.101.6.1644.

Lefering, R.; Huber-Wagner, S.; Nienaber, U.; Maegele, M.; Bouillon, B. Update of the trauma risk adjustment model of the TraumaRegister DGU: the Revised Injury Severity Classification, version II. *Crit Care* **2014**, *18*, 476, doi:10.1186/s13054-014-0476-2.

van der Sluijs, R.; van Rein, E.A.J.; Wijnand, J.G.J.; Leenen, L.P.H.; van Heijl, M. Accuracy of Pediatric Trauma Field Triage: A Systematic Review. *JAMA surgery* **2018**, *153*, 671-676, doi:10.1001/jamasurg.2018.1050.

Sherman, H.F.; Landry, V.L.; Jones, L.M. Should Level I trauma centers be rated NC-17? *J Trauma* **2001**, *50*, 784-791, doi:10.1097/00005373-200105000-00003.

Finally, Reference 7 was incorrectly given as Reference 9.

Bläsius, F. M. et al. DGU®, T. Helicopter emergency medical service and hospital treatment levels affect survival in pediatric trauma patients. J. Clin. Med. 10, 837 (2021).

Consequently, References have been renumbered accordingly.

The original Article has been corrected.

